# 845. Phosphate Derangements After Burn Injury: Clinical Impact of the Often-forgotten Electrolyte

**DOI:** 10.1093/jbcr/irag033.315

**Published:** 2026-04-06

**Authors:** Ryan D Rosen, Ryan T Davis, Silvia Aluia, Meredith Hazelrigg, Alfredo E Munoz-Laroche, Basel Mhaimeed, Heather Dolman, Samantha Tarras, Michael T White

**Affiliations:** Detroit Receiving Hospital Burn Center, Ferndale, MI; Wayne State University School of Medicine, Children's Hospital of Michigan Burn Center, Detroit, MI; Detroit Receiving Hospital Burn Center, Detroit, MI; Detroit Receiving Hospital Burn Center, Detroit, MI; Detroit Receiving Hospital Burn Center, Detroit, MI; Wayne State University School of Medicine, Detroit, MI; Detroit Receiving Hospital Burn Center, Detroit, MI; Indiana University Health, Indianapolis, IN; Detroit Receiving Hospital Burn Center, Detroit, MI

## Abstract

**Introduction:**

Phosphate derangements are common after severe burns; hyperphosphatemia results from tissue destruction, while hypophosphatemia occurs due to metabolic demand, intracellular shifting, and renal/exudative losses. Prior studies have linked hyperphosphatemia to increased mortality in burns, while findings for hypophosphatemia are mixed. As phosphate is filtered in the renal glomerulus, we propose that hyperphosphatemia signals renal dysfunction and risk for mortality, whereas hypophosphatemia does not predict adverse outcomes.

**Methods:**

We retrospectively reviewed Burn ICU admissions at a single American Burn Association-verified center from January 2013 to December 2022. Patients with pre-existing renal dysfunction were excluded. Laboratory data was collected from admission through hospital day 14 (HD14). Hyperphosphatemia was defined as serum phosphate ≥4.5 mg/dL, hypophosphatemia as ≤2.5 mg/dL. Significance was set at *p*<.05.

**Results:**

Among 687 patients, 86.5% had phosphate derangements - 36.8% hyperphosphatemia, 76.7% hypophosphatemia, and 27.1% both. Hyperphosphatemia was more common with larger burns (57.4% with TBSA >40% vs. 33.5% with TBSA <20%, *p*<.001); hypophosphatemia was not (*p*=.491). Overall mean phosphate declined from presentation to HD3 (3.61 to 2.77 mg/dL, *p*<.001), but was unchanged (4.73 to 4.39 mg/dL, *p*=.223) in patients requiring renal replacement therapy (RRT). Median initiation of RRT was HD5 (IQR 3-10), and was associated with increased risk for mortality (OR 9.59, *p*<.001).

Hyperphosphatemia on admission (OR 2.80, *p*=.013), HD2 (OR 9.80, *p*<.001) and HD3 (OR 44.11, *p*<.001) was independently associated with RRT, controlling for age, injury severity, and comorbidities. Hyperphosphatemia also predicted mortality (OR 2.24, *p*=.010), independent of RRT initiation. Hypophosphatemia was most prevalent on HD3, and was associated with reduced risk for RRT (OR 0.12, *p*<.001) and mortality (OR 0.48, *p*=.005); the association with mortality was lost after inclusion of RRT in the model (*p*=.430).

**Conclusions:**

In our study, hyperphosphatemia was associated with larger TBSA and increased risk for RRT and mortality. Admission hyperphosphatemia likely reflects injury severity (increasing risk for RRT and mortality), while persistent hyperphosphatemia represents impaired renal clearance and even greater risk for RRT. In contrast, hypophosphatemia was associated with a lower risk for RRT initiation, but not mortality; hypophosphatemia is likely a physiologic response to injury and resuscitation, and reflects preserved renal function.

**Applicability of Research to Practice:**

This study demonstrates an association between serum phosphate levels and outcomes after burn injury; monitoring serum phosphate levels should be considered as a cheap and readily available biomarker for renal failure and mortality after severe burns.

**Funding for the study:**

N/A.

